# Clinical Evaluation of Medical Ozone Use in Domestic Feline Cutaneous Wounds—A Short Case Series

**DOI:** 10.3390/ani13172796

**Published:** 2023-09-02

**Authors:** Nicuşor-Valentin Oros, Călin Repciuc, Ciprian Ober, Cosmin Peștean, Mircea Valerian Mircean, Liviu-Ioan Oana

**Affiliations:** 1 Department of Surgery, Faculty of Veterinary Medicine, University of Agricultural Sciences and Veterinary Medicine, 400372 Cluj-Napoca, Romania; nicusor-valentin.oros@usamvcluj.ro (N.-V.O.); ciprian.ober@usamvcluj.ro (C.O.); cosmin.pestean@usamvcluj.ro (C.P.); liviu.oana@usamvcluj.ro (L.-I.O.); 2 Department of Internal Medicine, Faculty of Veterinary Medicine, University of Agricultural Sciences and Veterinary Medicine, 400372 Cluj-Napoca, Romania; mircea.mircean@usamvcluj.ro

**Keywords:** cat, skin wounds, ozone treatment, second intention

## Abstract

**Simple Summary:**

Cutaneous wounds in cats are a common problem in veterinary practice as well as their therapy. Wound healing is a physiological process mediated by numerous growth factors that are successively released in order to restore the integrity of the skin. A longer time needed for wound healing in cats entails an increased risk of complications as well as higher total therapy costs. Ozone, used as a therapeutic complementary with regenerative properties, has begun to have wider applicability in veterinary medicine. Thus, the aim of this study was to clinically assess this as a therapeutic element in supporting cutaneous wound second-intention healing in cats. According to our knowledge, this is the first preliminary controlled study of its kind in cats.

**Abstract:**

Support and management of second-intention wound healing involves frequent dressing changes having different properties. Dressings can range from simple ones, such as nonadherent dressings, to more complex options, such as foam, hydrocolloid, alginate or negative pressure dressings. Seven cats were enrolled in the study with a total of nine wounds of various sizes with different etiology sizes and localizations. Three methods of local ozone administration were used to cover more of the ozone properties in the treatment of wounds: bagging, perilesional subcutaneous infiltrations and lavages with ozonated saline. Evaluation of the healing process was performed by clinical observation and wound area measurements every seven days until the complete recovery of the patients. The results of this study should encourage clinicians to consider medical ozone as a new therapeutic approach with regenerative properties in the second-intention healing of cats presenting cutaneous wounds.

## 1. Introduction

The physiological process of cutaneous wound healing consists of three phases: the inflammatory phase, the proliferative phase (angiogenesis, granulation, re-epithelialization) and the remodeling phase (extracellular matrix remodeling (ECM)) [1]. The physiological evolution of these healing phases is closely related to the release of cytokines and growth factors, of which the most representative are platelet-derived growth factor (PDGF), fibroblast growth factor (FGF), vascular endothelial growth factor (VEGF) and transformed growth factor (TGF-β) [2]. Wound healing depends on several factors, such as etiology, microbial population, susceptibility to infection, localization, vascularization of the perilesional tissue, size of the defect, tension and mobility of wound edges and the general condition of the underlying tissue [3].

Ozone, used as a regenerative therapeutic element, is obtained from medical oxygen using ozone generators, resulting in an oxygen–ozone mixture in which the maximum permitted concentration of ozone varies between 0.5 and 5%, corresponding to a range of 1–80 μg/mL (microgram of ozone/milliliter of oxygen) [4,5].

The mechanism of action of ozone at the tissue level is carried out in a direct and indirect way. In order for the tissues to benefit from the direct action of ozone, they must be exposed directly to an ozonated environment represented by a plastic bag filled with O3, ozonated water, ozonated saline or ozonated oils. Topical administration of ozone at high concentrations of 60–80 μg/mL has a good demonstrated bactericidal effect. This effect of ozone was noted during World War I when it was used to treat German soldiers suffering from gaseous gangrene as a result of an anaerobic infection caused by *Clostridium* spp. [6].

The bactericidal effect is supported by the hypothesis according to which this property of ozone is given by the action of free radicals. Its important antibacterial properties are manifested through the destruction of the bacterial cell membrane as a result of its fixation at the level of unsaturated bonds of phospholipids [7,8]. Ozone is also a naturally occurring molecule produced by neutrophils activated during antibacterial activity [9].

At concentrations lower than 20–40 μg/mL, ozone acts as a bioregulator [10]. Hydrogen peroxide (H_2_O_2_), at biological concentrations, is one of the most important bioregulators of oxidative stress [11].

Ozone has a high solubility and once in contact with the plasma, it reacts with various biomolecules, forming reactive oxygen species (ROS) and lipid ozonation products (LOP). They exert their action on cells in the body, which subsequently release various factors, promoting tissue regeneration [12]. H_2_O_2_ resulting from the reaction between ozone and polyunsaturated fatty acids at biological concentrations is one of the most important bioregulators of oxidative stress [11]. H_2_O_2_ is a fine-regulating mediator, with a proinflammatory role aimed at removing pathogens and exerting an anti-inflammatory role that prevents sustained inflammatory processes [12]. H_2_O_2_ increases the expression of 2,3-diphosphoglycerate at the level of erythrocytes, with favorable consequences in the transport and release of oxygen at the tissue level [13]. Exposure of human whole blood to ozone at concentrations of 22–156 μg/mL resulted in an increased release of TGF-β1 [14]. Valacchi and Bocci, in 1999, demonstrated the increased release of PDGF and TGF-β1 from platelets subjected to the ozonization process [15]. In 2009, Kim et al. demonstrated in an experimental study on guinea pigs that topical application of ozonized oil increases the expression of growth factors PDGF, TGF and VEGF in induced wounds. Also, fibroblast proliferation and collagen production are improved [16]. In human patients with diabetic ulcers, medical ozone administration accelerates healing by increasing the expression of growth factors in the early stage of therapy [17].

The aim of this study was to clinically evaluate the efficiency and feasibility of ozone treatment in second-intention healing protocols of cutaneous wounds in cats.

## 2. Materials and Methods

### 2.1. Patients

The study was conducted on seven cat (*Felis catus*) patients with skin lesions with different etiologies in different regions of the body presented between June 2021 and September 2022 to the Surgery and Intensive Care Department of the Faculty of Veterinary Medicine in Cluj-Napoca, Romania, chronologically, with owners who agreed with multiple visits for ozone treatment sessions. We included extensive, complicated or chronic wounds and also patients who received other conventional treatments without any positive results. The patients who presented with mild lesions or with owners who declined this treatment for financial constraints or insufficient trust in complementary therapies were excluded. All the owners were informed about ozone treatment and written consent was obtained for all cases used for the present study. Clinical and laboratory examination, rapid tests for detecting antibodies against feline immunodeficiency virus (FIV) and feline leukemia virus (FeLV) antigens (FIV Ab/FeLV Ag, VetExpert, United Kingdom) were performed on all patients, as well as the correction of homeostasis imbalances. Also, patients were surgical evaluated and debrided of devitalized tissues and microbiological samples for microbiological examination and antibiogram were taken (Table 1).

### 2.2. Ozone Preparation and Administration

**Figure 1:**
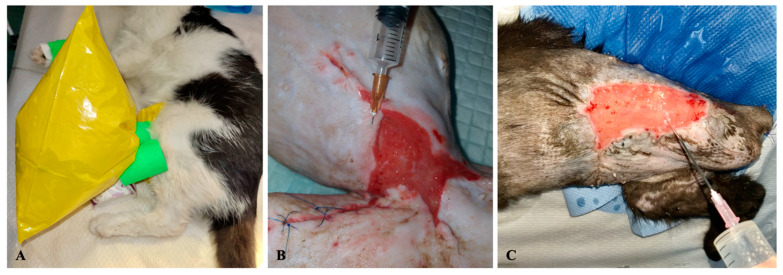
Methods of medical ozone administration used in our studies ((**A**)—plastic bag method, (**B**)—perilesional subcutaneous infiltration method, (**C**)–lavage with ozonized solution).

Medical ozone administration was performed in accordance with the Madrid Declaration of Ozonetherapy from 2020 and the WFOT’s Review on Evidence Based Ozone Therapy from 2015 [5,18] using three methods together: the bagging method (Figure 1A), the subcutaneous infiltration method (Figure 1B) and lavage with ozonized 0.9% saline solution (Figure 1C).

The ozone was produced using a medical generator (Medozon Compact; Herrmann Apparatebau GmbH, Elsenfeld, Germany), and the treatment was performed also by using a medical oxygen tube, extension iv. lines, polyethylene bags, vetrap, syringes of 2 mL, 20 mL, dermal needles 26 G and 18 G and a glass bubbler for the preparation of ozonized water (Aquazon; Herrmann Apparatebau GmbH, Germany). The lavage solution was 0.9% ozonized sodium chloride prepared prior to application using a gas concentration of 60 μg/mL bubbled for 20 min. After each session of medical ozone administration, the wounds were covered with a simple dressing so the evaluation of ozone benefits would not be influenced by the similar effects of other substances.

The number of sessions and the method of application were similar in each patient during the healing of skin defects (Table 2). The concentration of ozone used and the exposure time were variable, depending on the evolution of the wound healing.

### 2.3. Methods of Evaluation

**Figure 2:**
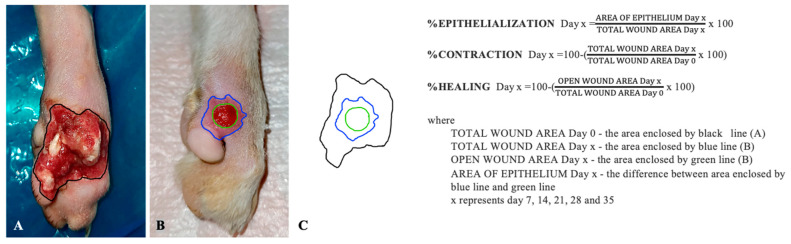
The formula for epithelialization, contraction and healing evaluation. (**A**) Total wound area in day 0—the area enclosed by black line; (**B**) Total wound area in day x—the area enclosed by blue line; Open wound area day x—the area enclosed by green line; (**C**) Area covered by new epithelium in day x—the difference between area enclosed by blue line and green line; x represents day 7, 14, 21, 28 and 35.

At the time of the first ozone treatment session, all wounds were evaluated photographically and their total surface was measured. A transparent foil and a thin-tipped marker were used to copy the contour of the wounds followed by transcription onto graph paper in order to establish the wound surface. All measurements were performed by the same person. The epithelialization, contraction and total wound healing speed were evaluated consecutively every seven days until complete healing of the skin defects. The clinical course was also assessed photographically using the same time frame. The three parameters were calculated according to the formula used by Bohling et al. in 2006 in a cutaneous defect study (Figure 2) [19].

### 2.4. Statistical Analysis

All data were reported as mean ± STDEV. Data were statistically analyzed with a paired Student’s T test and only *p* values < 0.05 were reported as relevant. (*p* < 0.05, >0.01—relevant; *p* < 0.01, >0.001—very relevant; *p* < 0.001—highly relevant; *p* > 0.05—irrelevant). The contraction, healing and epithelialization percentages obtained for days 7, 14, 21, 28 and 35 were reported compared to the percentage obtained for the previous treatment session. For day 0, the percentage was considered 0%.

## 3. Results

### 3.1. Clinical Evaluation

**Figure 3:**
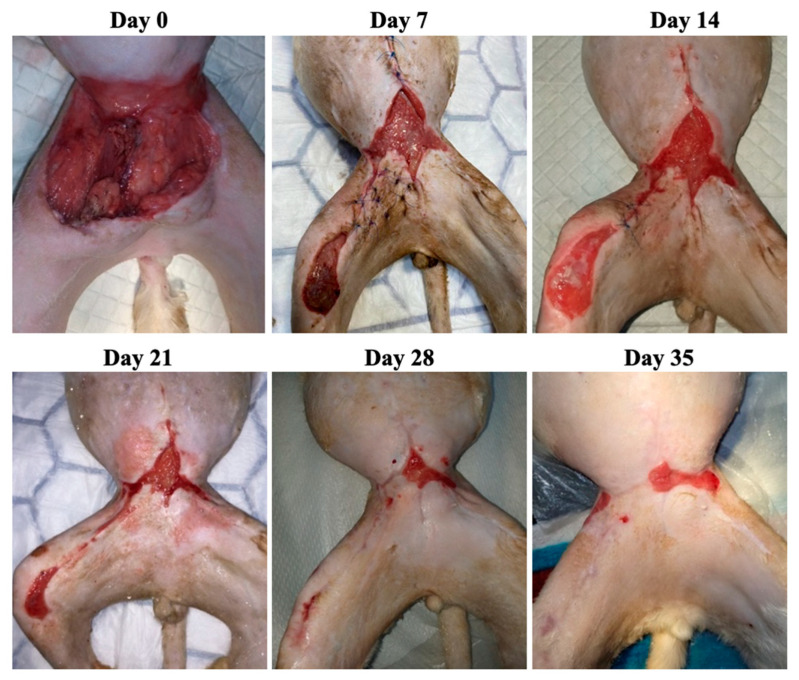
Case 1—Wound healing evolution.

Case 1 was represented by an 18-year-old male cat presenting a chronic, atonic wound in the caudal ventral and inguinal area (Figure 3). The patient was treated with antibiotic ointment for a few days without any results. After surgical evaluation, it was decided to try to reduce the defect by performing reconstructive surgery. This procedure resulted in two wounds measuring 20.75 cm^2^ and 9.17 cm^2^, respectively. The result of the FiV-FeLv rapid test was positive and the bacteriological examination from the wound exudates revealed *Streptococcus* spp. alpha-hemolytic, *Staphylococcus* spp., *Bacillus* spp. and Gram-negative (G−) bacilli sensitive to marbofloxacin and doxycycline. Marbofloxacin 4 mg/kg orally (PO) (Efex 10 mg, Ceva Santé Animale, Libourne, France) was used once daily for 5 days.

**Figure 4:**
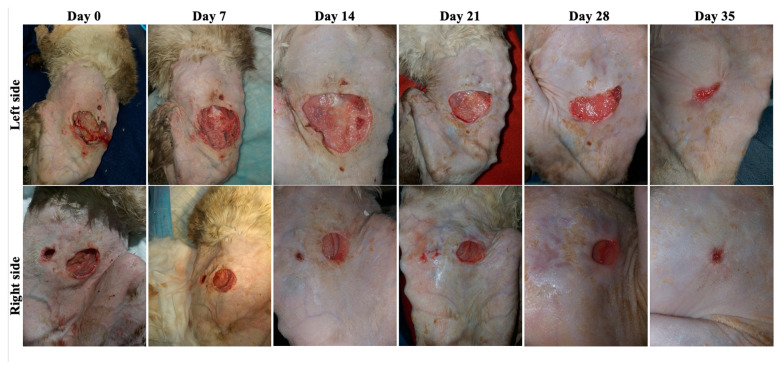
Case 2—Wound healing evolution.

Case 2 was represented by a 1-year-old female cat, presented with septic shock with an extensive wound at the level of the left thoracoabdominal area and tail avulsion (Figure 4). After stabilization, following further investigations (radiological and neurological examination), it was decided to amputate the left hind limb due to the fact that the distal extremity vascularization and functionality were totally compromised. The amputation resulted in a wound with an area of 29.74 cm^2^. Bacteriological examination revealed multiresistant Pseudomonas aeruginosa, *Staphylococcus* spp., *Enterococcus* spp., G-lactose-positive (coliform) and cocobacilli sensitive to enrofloxacin and amikacin were also identified. Amikacine 20 mg/kg intravenously (IV) (Amikozit 500 mg/2 mL, Zentiva, Bucharest, Romania) once daily and metronidazole 15 mg/kg IV (Metronidazole Braun 5 mg/mL, B. Braun, Melsungen AG, Germany) twice daily were used.

**Figure 5:**
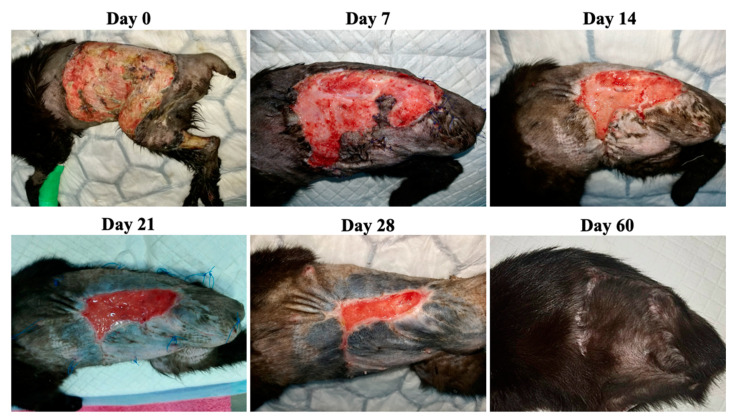
Case 3—Wound healing evolution.

Case 3 was a 14-year-old male cat presented with two purulent wounds on the sides of the abdomen as a result of a dog bite (Figure 5). The result of the FiV-FeLv rapid test was positive and the bacteriological examination revealed the presence of *Streptococcus* spp. and G-bacteria. Amoxicillin + clavulanic acid 15 mg/kg PO (Synulox 50 mg, Zoetis Belgium SA, Louvain-la-Neuve, Begia) twice per day for 5 days was used.

**Figure 6:**
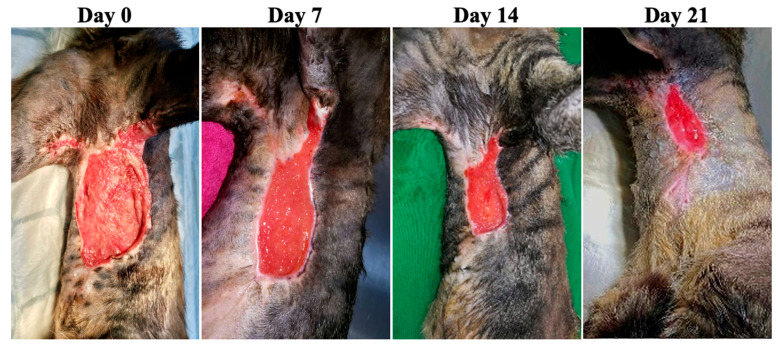
Case 4—Wound healing evolution.

Case 4 involved a 4-year-old male, castrated cat sent for evaluation of a chronic wound that had not responded to conventional therapy (Figure 6). Following surgical evaluation, the debridement of the wound edges was decided because of the presence of an excessive fibrous scar. After debridement, a wound of 35.25 cm^2^ was obtained. The result of the FiV-FeLV rapid test was positive for this patient as well and the bacteriological examination was negative. No antibiotic was used during ozone treatment.

**Figure 7:**
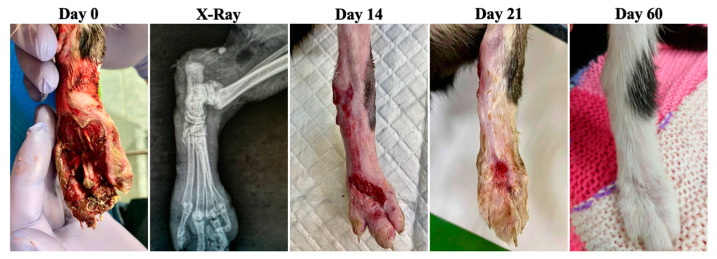
Case 5—Wound healing evolution.

Case 5 was represented by a 2-year-old female cat with a wound at the level of the medial region of the right tibio-tarso-metatarsal joint (Figure 7). Following radiological and surgical examination, a subluxation and an open wound with torn medial collateral ligaments of the joint were observed. Bacteriological examination revealed Escherichia coli sensitive to enrofloxacin. Enrofloxacin 5 mg/kg PO (Enroxil Flavour 15 mg, Krka, Novo Mesto, Slovenia) was used once daily for 5 days and metronidazole 15 mg/kg IV (Metronidazole Braun 5 mg/mL, B. Braun, Melsungen AG, Germany) 2x daily for 5 days.

**Figure 8:**
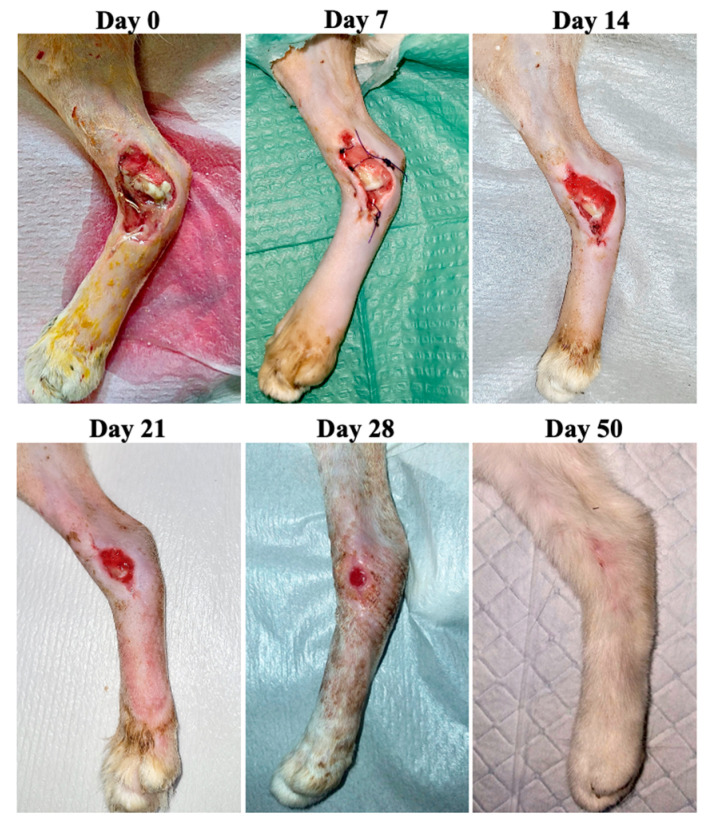
Case 6—Wound healing evolution.

Case 6 was represented by a 6-year-old male cat with an acute wound at the level of the left hind limb at the medial tibial region caused by a car accident (Figure 8). Radiologically, the following aspects were observed: distal fracture of the left tibia and multiple comminuted fractures of the phalanx. It was decided to stabilize the tibial fracture, as well as amputate the phalanges of the third finger. The primary closure of the phalangeal wound was impossible so a second intention of healing of the 4.7 cm^2^ wound was performed. The bacteriological examination was negative and no antibiotic was used before or during ozone treatment.

**Figure 9:**
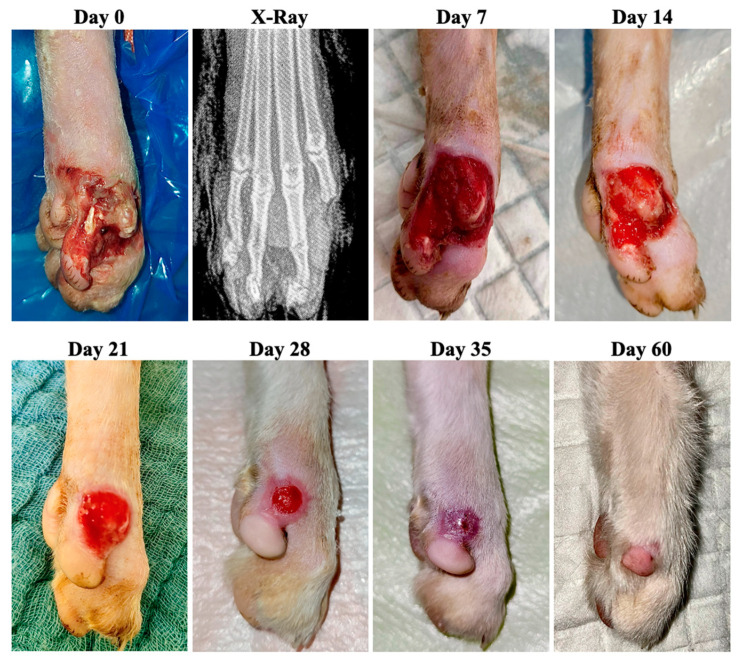
Case 7—Wound healing evolution.

Case 7 was represented by a 2-year-old female cat with a wound measuring 6.21 cm^2^ at the medial extremity of the left hind limb with an unknown cause (Figure 9). Radiologically, the avulsion of the second and third phalanx of the second finger was observed. Bacteriological examination revealed Escherichia coli sensitive to amoxicillin + clavulanic acid. Amoxicillin + clavulanic acid at a dose of 15 mg/kg PO (Synulox 50 mg, Zoetis Belgium SA, Louvain-la-Neuve, Belgium) twice per day for 5 days was used.

### 3.2. Planimetry

#### Epithelialization, Contraction and Healing

The most significant percentage of epithelialization was observed between day 21 and day 28 (*p* = 0.001) with a mean epithelialization percentage of 68.652 ± 19.935%. The average percentage of the contraction process was 16.031 ± 7.954% on day 7, the most significant percentage of contraction being observed between day 0 and day 7 (*p* = 0.0002). Between day 14 and day 21, the most significant healing rate was observed (*p* = 0.0006) with an average percentage of 72.985 ± 13.269 for day 21. After 35 days of therapy, the healing percentage of the wounds was more than 98% (Table 3).

## 4. Discussion

The total healing time needed for cat wounds is longer than in dogs because during second-intention wound healing, the contraction phase predominates, unlike in dogs, where healing is based on the proliferative phase of granulation and epithelialization [19]. All stages of wound healing are controlled by redox processes [20].

Ozone is known to have high oxidative effects due to its hydrogen peroxide production in contact with biological fluids. However, in agreed medical concentrations, it induces a mild transitory oxidative stress that will produce a mild stimulation of the antioxidant system of the animal without overwhelming it. All of these have been shown in studies performed on human blood ozonized with concentrations between 20 and 80 μg/mL 

Currently, there are no clinical trials to demonstrate that ozone treatment methods are superior to other therapies in the management of wounds in cats [13].

In terms of wound care, saline lavage remains a standard component of therapy with a role in mechanical cleaning and reducing bacterial load. A systematic review of ozonated topical liquids claims that their use in both veterinary and human patients reduces total wound healing time. The same authors encourage the use of ozonated liquids as part of general wound healing therapies [21]. Ozone, in therapeutic concentrations, intervenes in redox processes accelerating them when used in topical applications like ozonized water in the management of induced wounds in Wistar rats [22].

The ozonized saline solution improved the healing of lesions produced in vitro in keratinocyte cell cultures stimulating the cell proliferation processes. This effect has been attributed to 4-hydroxynonenal (HNE) and H_2_O_2_, molecules produced by contact between ozone and the biological substrate [23]. Once the skin tissue is damaged, dermal fibroblasts near the lesion migrate and proliferate in the damaged area, producing an extracellular matrix, and then they change their phenotype into myofibroblasts. They later attach via pseudopods to the collagen and fibronectin components in the extracellular matrix, stimulating the contraction phase of the wound [24].

Ozone administered topically in the treatment of induced oral wounds in rats has been shown to be effective in advancing healing and improving angiogenesis and fibroblast density [22]. Ozonated fluids inhibit the activity of nuclear factor kappa B (NF-kB), suggesting the idea that these formulas have anti-inflammatory and immunomodulatory roles [25].

Collagen synthesis during the evolution of wound healing depends on fibroblast activity, with nitric oxide playing an essential role in this process [12]. The beneficial activity of ozone in the wound epithelialization phase may be due to ozone’s ability to release nitric oxide and produce prostacyclin [26,27].

Exposure of nonactivated fibroblasts of human origin at doses of 10–30 μg/mL O_3_ stimulates proliferation, formation of cell surface protrusions and an increased expression of IL-6 and TGF-β1 [28].

Other studies support the superior benefits of ozone treatment in wound healing compared to other conventional methods of therapy due to ozone’s ability to stimulate angiogenesis, fibroblast activity and collagen production [16,29,30]. Blood exposure to ozone increases the erythrocyte glycolysis rate by stimulating the release of 2,3-diphosphoglycerate [31]; this is supported by studies evaluating the effect of ozone on distal limb ulcers in human diabetic patients, demonstrating that this therapy increases the expression of VEGF, PDGF and TGF-β [17]. Wainstein et al. in 2011 also reported ozone treatment being superior to conventional therapies in the healing process of diabetic foot ulcers [32].

Perilesional subcutaneous administration of ozone reduces pain through the direct oxidation of algogenic mediators and receptors [33]. Moreover, a single subcutaneous administration of ozone decreased allodynia, caspase expression and IL-1β immunoreactivity in orbitofrontal cortex astrocytes in mice with induced neuropathy [34]. It also decreases the pruriginous sensation by inhibiting prurinergic receptors P2X3 and P2X7 [35]. These properties of ozone improve comfort through pain reduction. The treatment sessions were well tolerated by the patients included in the study, speeding up the patients’ recovery.

A systematic review that evaluated the therapeutic potential of ozone in human chronic wounds concluded that ozone treatment proved itself to be effective with encouraging results and compared to standard treatments, it can shorten healing times, but further research is needed in this direction [36]. Our treatment protocol included the constant application of ozone in progressively lower concentrations. Based on the hormetic action of ozone, 60 micrograms/mL were used for antibacterial purposes and concentrations below 10–30 micrograms/mL were used for their bioregulatory effects [7]. Al-Saadi H. et al. in 2015 mentioned a 99% efficiency of ozonized saline solution against planktonic Staphylococcus aureus [37]. This antibacterial effect of ozone is also clinically visible in our study, with some subjects receiving antimicrobial therapy only in the first 5–10 days of treatment. Clinical evaluation of this aspect supports the antimicrobial activity of ozone. Future research should evaluate the exact effectiveness of topical ozone treatment on bacterial wound clearance in animals. Ozone treatment administered through subcutaneous infiltration and plastic bag methods proved effective in accelerating the rate of skin graft acceptance, with antibiotic therapy being used only in the first 5 days [38]. Histologically, wounds treated by ozone subcutaneous perilesional infiltrations presented fewer inflammatory cells and a greater number of structural fusiform cells in the newly formed granulation tissue [39].

In this context, Repciuc C. et al. in 2020 considered ozone treatment as an alternative in the postamputation case of a cat diagnosed with FIV^+^ with wound dehiscence that did not respond at all to conventional therapies [40].

A comparative study of wound healing induced in dogs and cats obtained similar results in epithelialization, healing and contraction speed on days 7, 14 and 21 but without the use of complementary supportive therapies. In this study, the percentage of epithelialization was 0, 13 and 34 compared to our results of 4.58, 12.04 and 33.38, and the percentage of contraction was 18.2, 53.0 and 75.8 compared to our results of 16.03, 36.99 and 61.73 and the percentage of total healing was 18.3, 59.0 and 83.9 compared to our results 19.71, 44.15 and 72.98 [41]. It is worth mentioning that the wounds in this study were experimentally induced under conditions of asepsy, not being subjected to infectious processes and various pathogenic populations.

A limitation of the present study is represented by the absence of a control group in which wound therapy would have not been performed, this being considered unethical according to the law as well as the authors. Also, the values obtained after the evaluation of the wound area are heterogeneous due to the variability of wound sizes, location and the immune status of patients taken in the study.

The costs of this treatment are relatively low, the major investment being represented by the ozone generator. The expenses of each session of treatment are represented only by the usual materials used daily in veterinary clinics consisting of needles, syringes, polypropylene bags, infusion extensions and a source of pure medical oxygen.

In the end, by using the three methods of administration together, we might attain a synergistic effect [18]. The physiologic saline solution acts as a lavage element and has the purpose of reducing the superficial bacterial charge of the wound as it also has immunomodulating, anti-inflammatory and proepithelializing properties. The bagging technique might help the oxygen delivery to the tissues [13], control the infectious processes and stimulate the platelet-derived growth factors release (aspects that promote healing) and the perilesional subcutaneous infiltrations have local anti-inflammatory and analgesic effects as ozone has the capacity to oxidize pain receptors. Considering all these aspects, the three-method ozone wound treatment might cover all the needs of a wound under therapy but this hypothesis needs further research as well as evaluating their individual medical benefits. These positive clinical results encourage new prospective controlled studies with multiple groups and supplementary methods of evaluation for even more complex results. Comparing these methods of ozone treatment with other conventional treatments would be an interesting perspective as well.

## 5. Conclusions

The results of this study suggest that ozone used as a complementary treatment in secondary healing of wounds supports the recovery of patients with extensive acute and chronic wounds, even the immunosuppressed ones. This could be due to the ability of ozone to improve local circulation, increase the release of platelet growth factors and decrease the bacterial load in wounds if used according to the protocol described.

This preliminary study supports the fact that ozone treatment for secondary healing of cutaneous wounds in cats has positive effects and it also might be effective in patients suffering from feline immunodeficiency.

## Figures and Tables

**Table 1:** Characterization of patients included in the study, microbiological examination and antibiotics used.

Case	Breed	Sex	Weight (kg)	Age (Years)	Etiology	Affected Area	Associated Pathologies	Microbiological Examination	Sensibility	Antibiotics Used (Days)
1	Domestic Shorthair cat	M	4.2	18	Chronic wound	Abdomino-inguinal	- FIV+	- *Streptococcus* spp. alpha-hemolytic - *Staphylococcus* spp. - *Bacillus* spp. - *bacilli G*^−^	Marbofloxacin Doxycycline	Marbofloxacin (7)
2	Domestic Shorthair cat	F	2.1	1	Road accident	Left abdominal side	- Severe regenerative anemia - Sepsis	- *Pseudomonas aeruginosa* (multiresistant) - *Staphylococcus* spp. - *Enterococcus* spp. - *Cocobacilli G*^−^ - lactose-positive (coliform)	Enrofloxacin Amikacin	Amikacin (7) Metronidazole (10)
3	Birman cat	M	1.9	14	Bitten by a dog	Both sides of the abdomen	- FIV+ - Bilateral phlegmon	- *bacilli G*^−^ - *Streptococcus* spp.	Amoxicillin + Clavulanic acid Cephalexin Enrofloxacin	Amoxicillin + Clavulanic acid (5)
4	Domestic Shorthair cat	M	2.7	4	Hit by car	Ventral part of the abdomen	- FIV+	-	-	-
5	Domestic Shorthair cat	F	3.1	2	Unknown	Right hind limb Tibio-tarso-metatarsal region	- Subluxation - Digital joint extensor and compromised collateral ligaments	- *Escherichia coli*	Enrofloxacin	Enrofloxacin (5) Metronidazole (7)
6	Domestic Shorthair cat	M	3.6	6	Hit by car	Left hind limb medial part	- Distal tibia fracture affected limb - Cominutive fractures at the level of phalanges II, III and IV	-	-	-
7	Domestic Shorthair cat	M	4.1	2	Unknown	Left hind limb medial part	- Avulsion of phalanges II and III of the finger 2	*Streptococcus* spp.	Amoxicillin + Clavulanic acid	Amoxicillin + Clavulanic acid (5)

**Table 2:** Method of application of ozone treatment and number of sessions.

Case	Method	Number of Treatment Sessions
1	- bagging conc. 20–60 μg/mL for 5–20 min	12
	- perilesional infiltration with 15 μg/mL	6
	- lavage with ozonized saline solution at 60 μg/mL	12
2	- bagging conc. 20–60 μg/mL for 5–20 min	16
	- perilesional infiltration with 15 μg/mL	6
	- lavage with ozonized saline solution at 60 μg/mL	16
3	- bagging conc. 20–60 μg/mL for 5–20 min	12
	- perilesional infiltration with 15 μg/mL	6
	- lavage with ozonized saline solution at 60 μg/mL	12
4	- bagging conc. 20–60 μg/mL for 5–20 min	8
	- perilesional infiltration with 15 μg/mL	6
	- lavage with ozonized saline solution at 60 μg/mL	8
5	- bagging conc. 20–60 μg/mL for 5–20 min	10
	- perilesional infiltration with 15 μg/mL	6
	- lavage with ozonized saline solution at 60 μg/mL	10
6	- bagging conc. 20–60 μg/mL for 5–20 min	8
	- lavage with ozonized saline solution at 60 μg/mL	8
7	- bagging conc. 20–60 μg/mL for 5–20 min	10
	- perilesional infiltration with 15 μg/mL	4
	- lavage with ozonized saline solution at 60 μg/mL	10

**Table 3:** Evolution of the contraction, total healing and epithelialization mean ± standard deviation expressed in percentages reported to the previous session of therapy on days 7, 14, 21, 28 and 35 of treatment. * Indicates significance with *p* ≤ 0.001.

Planimetry	Day					
	0	7	14	21	28	35
Contraction %	0	16.031 ± 7.954 *	36.998 ± 15.702	61.734 ± 13.269	79.482 ± 11.844	89.747 ± 9.880
Healing %	0	19.714 ± 9.626	44.156 ± 15.262	72.985 ± 13.269 *	91.960 ± 7.222	98.193 ± 2.066
Epithelialization %	0	4.583 ± 3.678	12.045 ± 4.902	33.387 ± 18.247	68.652 ± 19.935 *	89.104 ± 13.606

## Data Availability

Additional data of this study are available from the main author.
